# Environmental and Human Health Impacts of Agricultural Pesticides on BIPOC Communities in the United States: A Review from an Environmental Justice Perspective

**DOI:** 10.3390/ijerph22111683

**Published:** 2025-11-06

**Authors:** Belay Tizazu Mengistie, Ram L. Ray, Ayodeji Iyanda

**Affiliations:** 1 College of Agriculture, Food and Natural Resources, Prairie View A&M University, Prairie View, TX 77446, USA; raray@pvamu.edu; 2 Division of Social Science, College of Arts and Science, Prairie View A&M University, Prairie View, TX 77446, USA

**Keywords:** pesticide exposure, environmental justice, human health risks, environmental impacts, BIPOC, disproportionate, USA

## Abstract

In recent years, public discourse on pesticide impacts has increasingly recognized institutional and structural racism as key drivers of health disparities in Black, Indigenous, and People of Color (BIPOC) communities. While pesticides are vital for crop protection from causing yield losses, extensive research highlights their adverse effects on environmental quality and human health. These impacts disproportionately burden BIPOC populations, making pesticides a major environmental justice (EJ) concern like many other environmental pollutants. Despite progress in understanding these effects and advancing EJ, significant technical, social, and policy gaps remain. The objective of this review is to systematically examine critical gaps in technical, social, and policy dimensions, as well as the environmental and human health impacts of pesticide exposure on BIPOC communities in the United States, through the lens of environmental justice. This review synthesizes 128 sources peer-reviewed articles, books, reports on pesticides, EJ, and BIPOC communities in the U.S. Key findings reveal uneven distribution of pesticide-related health and environmental burdens along racial, ethnic, and socioeconomic lines. Non-Hispanic Blacks and Mexican Americans exhibit higher pesticide biomarkers and greater exposure risks than non-Hispanic Whites. Structural racism and classism, rooted in historical systems, perpetuate these inequities, compounded by regulatory failures and power imbalances. In addition, the EPA has flagged 31 pesticide manufacturing facilities for “Significant Violations” of key environmental laws, including the Clean Air Act, Clean Water Act, and Resource Conservation and Recovery Act. These systemic issues underscore urgent needs for transparency, accountability, and equitable policy reform. An EJ framework exposes critical knowledge gaps and calls for structural changes to ensure equal protection and responsive policies for the most affected communities.

## 1. Introduction

In recent years, public discourse has increasingly foregrounded institutional and structural racism as critical determinants of the well-being of Black, Indigenous, and People of Color (BIPOC) communities. Environmental toxicity, particularly exposure to harmful pesticides intersects with these systemic inequities, collectively shaping both the immediate and long-term health outcomes of BIPOC individuals across the lifespan [1,2]. Pesticides are widely used in modern agriculture as a cost-effective means to boost crop yield and quality, playing a vital role in ensuring global food security amid a growing population. Pesticides as agricultural technologies designed to prevent, destroy, repel, or mitigate pests and weeds [1,2,3] encompass a broad range of substances including insecticides, herbicides, fungicides, bactericides, and rodenticides. The prevalence of pests, weeds, and diseases in agricultural systems has been a major factor behind the widespread adoption of pesticides and, among others, formed one of the driving forces behind the Green Revolution, especially since the 1930s [4]. Without the use of pesticides, estimated crop losses due to pest damage would reach 78% for fruits, 54% for vegetables, and 32% for cereals [4,5]. Additional benefits of pesticide use include extended shelf life of produce and reduced labor requirements for tasks such as weeding, thereby freeing up the workforce for other agricultural activities [5]. Despite advancements in agricultural science, crop losses due to pests and diseases still range from 10% to 90%, with an average of 35–40% across all major food and fiber crops. Combined with high-yield crop varieties, expanded cultivation, fertilizers, and soil amendments, pesticide use has played a key role in boosting agricultural productivity [5,6,7,8].

Today, there is broad recognition that all individuals regardless of race, color, national origin, or income deserve equal protection under environmental laws and equitable access to their benefits. Yet, despite this progress, a growing body of evidence reveals that communities of color and those with low incomes continue to bear a disproportionate burden of environmental harm, both in the United States and globally [9,10]. Specific events, policies, practices or regulations and laws shape developmental contexts that can be perpetuated across multiple generations. Historically or structurally racist policies, practices, or events can be both recent occurrences, e.g., the decision-making leading to the Flint, Michigan, water contamination crisis, or more distant practices, e.g., redlining [11].

Excessive pesticide use has profound environmental and human health consequences [12,13,14,15]. The application of agricultural pesticides poses significant risks to farm workers and nearby communities due to pesticide drift during spraying or fumigation, volatilization into the air, contamination of household dust, and direct exposure for agricultural workers and their families [8,16]. Pesticides can contaminate water sources, including drinking water, through runoff and infiltration [1,8]. Numerous studies [4,16,17,18,19], among others, have documented the harmful effects of indiscriminate agrochemical use on both ecosystems and human well-being. A global survey on pesticide management in agriculture revealed that over 20% of countries reported major incidents of environmental pollution caused by pesticides [7,20]. Workers involved in pesticide manufacturing, distribution, retail, and application are particularly vulnerable to direct exposure. Additionally, individuals living near agricultural fields are at risk [1,8,19]. In 17 Latin American countries, over one million tons of pesticides are used annually on crops, posing health risks to farm workers, their families, and nearby communities [15]. Across the United States, the entire lifecycle of pesticides from production to application disproportionately affects communities of color and those with low incomes. Over the past 50 years, the environmental justice movement has made significant strides [21,22]. Environmental justice (EJ) is the principle that all individuals and communities deserve equal protection under environmental and public health laws. The U.S. Environmental Protection Agency (EPA) further defines it as the fair treatment and meaningful involvement of all people regardless of race, color, national origin, or income in the development and enforcement of environmental policies [22].

Studies have consistently shown that racial and ethnic minority groups, along with low-income communities, experience disproportionately poorer environmental and health outcomes [18,21,22,23,24]. These communities are more frequently exposed to multiple environmental hazards and social stressors, including poverty, food insecurity, substandard housing, and systemic inequality. In the United States, nearly 90% of pesticide use occurs in the agricultural sector, placing farmworkers and their families, many of whom belong to marginalized communities, at heightened risk from these hazardous chemicals [18,25,26]. The history of agricultural labor in the US is deeply rooted in exploitative systems designed to uphold white supremacy and suppress the upward mobility of people of color. From the use of enslaved African labor on Southern plantations to sharecropping, indentured servitude, and the exploitation of Asian immigrants for low-wage farm work on the West Coast, these racist agrarian structures have shaped the foundations of modern agriculture [27,28,29].

The relationship between environmental justice and the health of farm workers and their communities underscores longstanding structural inequalities, which have been exacerbated by the expansion of the agro-industrial model and its resulting socio-environmental harms. Numerous studies including those by Bullard [15,21,22,30,31,32] along with analyses of U.S. Census data, reveal that Hispanic/Latino, African American, and Asian populations are disproportionately exposed to higher volumes of pesticides and greater toxicity-weighted pesticide use. Environmental pollutants, including pesticides, are known to disproportionately affect Black, Indigenous, and People of Color (BIPOC), as well as low-income and low-wealth communities. These disparities are deeply rooted in a complex history of systemic oppression, maintained through structural racism and classism in the United States [24,33,34,35]. A recent study conducted by Donley et al. [18] found that biomarkers for 12 harmful pesticides were present in the blood and urine of Black and Mexican Americans at levels up to five times higher than those found in White Americans. Additionally, an estimated 10,000 to 20,000 predominantly Latinx agricultural workers fall ill each year due to pesticide exposure, yet they remain excluded from many of the protections afforded to the general public [15,18].

Much of the existing research has focused on disparities based on race/ethnicity or socioeconomic status, while some studies have also explored how gender and geography interact with these factors. CDC biomonitoring data shows that Mexican American and African American women over 40 have significantly higher levels of pesticide metabolites in their blood and urine than white women [11,14,18,27]. However, most research lacks a unified theoretical framework and is often constrained by the availability of relevant data [18,36]. The structural mechanisms that perpetuate environmental injustices in the U.S. remain underexamined [30,37]. Given the well-documented risks that many agricultural pesticides pose to ecosystems and human health, and the fact that these risks are not evenly distributed, scholars and activists have increasingly framed pesticide exposure as an issue of EJ. This framing highlights how BIPOC, as well as low-income communities, are disproportionately burdened by pesticide-related harms. There is a growing volume of scientific literature on the adverse effects of agrochemical use on both environmental quality and human health. Despite ongoing debate, the EJ movement has shed light on the issue by emphasizing that marginalized communities are more likely to be exposed to environmental hazards. These reviewers understand that scholars have produced a substantial body of research on two key areas: (a) the nature of health disparities in the United States, and (b) the dynamics of environmental inequality. However, despite this progress, both bodies of literature remain limited in scope for several reasons. Additionally, significant knowledge gaps persist in advancing environmental justice as a meaningful solution for pollutants. Therefore, this review examines existing literature and available datasets to assess the extent of disparities in exposure and harm related to one of the world’s most pervasive pollutants: agricultural pesticides. This review identifies critical gaps in the technical, social, and legal dimensions of how agricultural pesticide use impacts BIPOC communities in the United States framed through the lens of EJ.

## 2. Methods and Approaches

This study employed a systematic literature review (SLR) in accordance with PRISMA 2020 guidelines to ensure transparency and rigor. This systematic review has been registered in PROSPERO (International Prospective Register of Systematic Reviews) and is publicly accessible in the registry at [https://www.crd.york.ac.uk/PROSPERO/register/TemplatePreview], accessed on 6 October 2025, under registration ID 1183914. The protocol outlines the objectives, methodology, and planned analyses to ensure transparency and reduce the risk of bias. The review focused exclusively on studies conducted within the United States to capture the development and underlying narratives related to environmental and human health impacts of agricultural pesticides on BIPOC communities from an environmental justice perspective. Similarly, Nielsen and D’haen highlighted the importance of clearly communicating qualitative research methods in interdisciplinary settings [38]. A comprehensive search was conducted across major academic databases and credible web sources, including PubMed, Scopus, Web of Science, ScienceDirect, and Google Scholar. Keywords such as “pesticide,” “environmental justice,” “environmental and human health impacts,” and variations of “Black, Indigenous, and People of Color (BIPOC)” were combined using Boolean operators (e.g., AND) to structure the search term. The strategy also incorporated hyphenated and non-hyphenated variations to maximize coverage.

**Figure 1:**
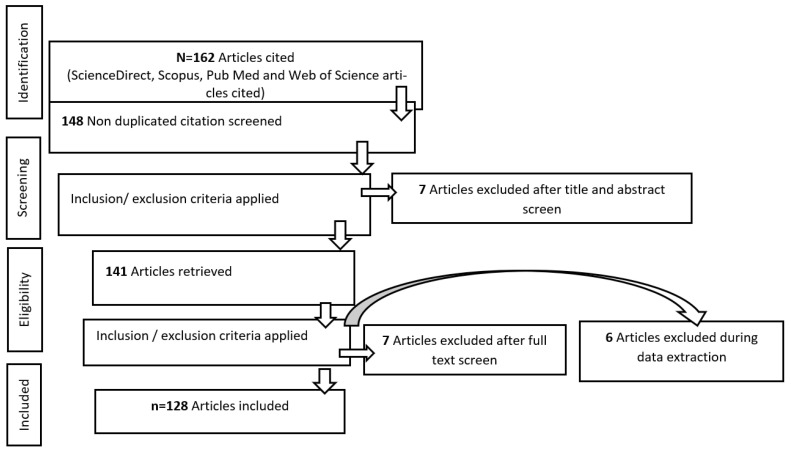
Procedures used for systematic literature reviews.

The article selection followed the standard PRISMA stages: identification, screening, eligibility, and inclusion/exclusion criteria (see Figure 1). Two reviewers independently screened titles, abstracts, and full texts to minimize bias. Discrepancies were resolved through consensus. Eligibility was based on inclusion/exclusion criteria. The inclusion criteria include studies addressing BIPOC communities in the U.S., agricultural pesticide exposure, and environmental or health outcomes whereas the exclusion criteria include non-U.S. studies; non-agricultural pesticide exposure; research focused on livestock management, indoor pest control, or disease vector control; and articles lacking environmental justice framing. We conducted a comprehensive systematic review between May and July 2025, covering studies from Bullard’s seminal 1983 work on environmental justice through the latest publications in 2025. This time frame aligns with both historical context and contemporary developments, enabling an analysis of temporal trends in environmental justice and public health research.

From an initial 162 records, duplicates were removed, leaving 141 articles for screening. Additional sources were identified through snowballing (reference checks) and gray literature searches, resulting in 128 studies included and cited in the final analysis. Data were synthesized thematically under key categories: *pesticides*, *health effects*, *environmental impacts*, *BIPOC communities*, and *environmental justice*. Emphasis was placed on U.S. based research published within the past decade to capture recent trends and policy implications. To complement the literature review, pesticide use data, toxicity profiles, and exposure information were gathered from authoritative sources, including U.S. Environmental Protection Agency (EPA), U.S. Census Bureau, Beyond Pesticides, Center for Biological Diversity (CBD), Pesticide Action Network North America (PAN North America), Pesticide-Induced Diseases Database, Centers for Disease Control and Prevention (CDC). These sources provided regulatory, demographic, and toxicological data to strengthen the contextual analysis.

## 3. Environmental Justice as a Conceptual Framework

As defined by Bullard [22] and Claudio [36], environmental justice is the principle that “*all people and communities are entitled to equal protection of environmental and public health laws and regulations.” The Environmental Protection Agency of the US (EPA-US) expanded on this definition in 1995, describing environmental justice as “the fair treatment and meaningful involvement of all people regardless of race, color, national origin, or income with respect to the development, implementation, and enforcement of environmental laws, regulations, and policies*”. According to the EPA, fair treatment means that no population should bear a disproportionate share of the negative human health or environmental consequences resulting from industrial, municipal, and commercial operations, or from the execution of federal, state, local, and tribal programs and policies especially due to policy or economic disempowerment [14,23,30,39].

Environmental justice is distinct from environmental inequality or environmental injustice which refers to situations where specific social groups are disproportionately affected by environmental hazards [32,40,41]. A particularly harmful form of environmental inequality is environmental racism. The term was first defined by Chavis, as cited in Brulle and Pellow [27], as: “*Racial discrimination in environmental policymaking, the enforcement of regulations and laws, the deliberate targeting of communities of color for toxic waste facilities, the official sanctioning of the life-threatening presence of poisons and pollutants in our communities, and the history of excluding people of color from leadership in the ecology movements*.” Environmental racism, therefore, refers to any policy, practice, or directive that intentionally or unintentionally differentially affects or disadvantages individuals, groups, or communities based on race or color [22,36,42,43]. This framing emphasizes the systemic nature of environmental harm and the need for justice-centered approaches to environmental governance.

Environmental injustice is a complex and deeply rooted issue that reflects the unequal distribution of environmental burdens and benefits across communities [25,43]. It goes beyond the mere presence of pollution or the absence of green spaces, it concerns how these environmental conditions disproportionately affect certain populations based on race, ethnicity, socioeconomic status, and geographic location. Understanding environmental injustice requires a critical examination of the power structures, policy decisions, and historical legacies that sustain these disparities. This article explores the multifaceted nature of environmental injustice and its profound consequences for affected communities. At its core, environmental injustice refers to the unfair and unequal allocation of environmental risks and resources. This manifests in several keyways: *(i) disproportionate exposure to pollution, (ii) limited access to clean air, water, and green spaces, (iii) unequal enforcement of environmental regulations, and (iv) restricted participation in environmental decision-making processes* [44,45].

The roots of environmental injustice are deeply embedded in historical systems of discrimination and exclusion [21,23,46]. In the United States, one prominent example is the practice of redlining, which systematically denied services such as housing loans and insurance to residents of certain neighborhoods based on race. This discriminatory policy led to the creation of communities that were deliberately underserved and often situated near industrial zones and pollution sources. The environmental health consequences of these policies continue to affect marginalized communities today. Similarly, colonial practices and the exploitation of Indigenous lands have long placed Native communities at the forefront of environmental degradation [47,48]. These communities have been disproportionately exposed to environmental hazards due to forced displacement, resource extraction, and the siting of polluting industries on or near tribal lands. Together, these historical injustices have laid the foundation for the environmental inequalities that persist today [32,35,49].

As a conceptual framework, EJ integrates both social and ecological concerns to examine how environmental stressors are unequally distributed across society [40,41]. EJ scholarship explicitly connects environmental issues often framed as “green” concerns to broader questions of class, race, gender, and social justice. It seeks to uncover when, how, and why marginalized groups and communities disproportionately bear the burden of environmental harm [41,50], often in close alignment with grassroots social movements [23,27]. EJ is inherently political, calling for mechanisms to assign accountability and to redress harm through targeted remedial actions and resource allocation [51]. Originating in North America, the concept is rooted in the struggles of low-income and minority communities exposed to hazardous land uses such as toxic waste sites and polluting industries [52,53]. Over the past few decades, however, EJ has proven analytically valuable across a wide range of global contexts [54,55,56] and has served as a powerful framework for community mobilization [57]. Influenced by theorists such as Fraser [58] and Young [59], EJ scholarship has evolved to encompass three interrelated dimensions of justice: distribution (of environmental benefits and burdens), recognition (of marginalized identities and experiences), and participation (in environmental decision-making) [23].Within the EJ literature on food and agriculture, agrochemicals particularly pesticides used in industrial-scale farming remain a central concern due to their disproportionate impacts on farm workers and nearby communities.

**Figure 2:**
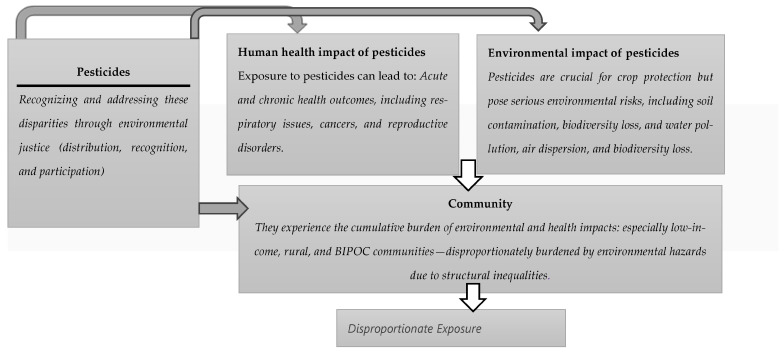
The interaction between pesticides, their effects on human health and the environment, and environmental justice as a potential remedy.

Figure 2 illustrates five interconnected domains at the core of this framework, highlighting their dynamic and reciprocal relationships. For instance, if pesticides are not regulated appropriately (in terms of production, distribution, use and disposal), they can have undesirable effects on the environment and human health. These impacts are not distributed equally and communities with the least political and economic power often bear the greatest risks. This framework emphasizes the importance of centering community voices, fostering resilience, and promoting participatory governance in environmental health decision-making. Guided by EJ as an analytical lens, we have reviewed our insights into thematic areas in the following section.

## 4. Literature Insights

### 4.1. The Environmental Justice (EJ) Movement

The EJ movement originated from the civil rights era of the 1960s–1970s, when activists recognized that marginalized communities faced disproportionate exposure to pollutants and environmental hazards. Some successful community-led events that fought environmental injustice occurred before the EJ movement came into action, including: The Memphis Sanitation Strike in Memphis, Tennessee; The Northeast Community Action Group (NECAG) in Houston, Texas; and Sit-in against Warren County Landfill [18,60]. In 1983, sociologist Robert Bullard noticed that garbage dump facilities in Houston, Texas, were disproportionately situated in African American neighborhoods. Despite African Americans comprising only 27.6% of the city’s population at the time, all five municipal landfills and four out of five trash incinerators were located in predominantly Black communities; the fifth incinerator was sited in a Hispanic neighborhood [21,22,25]. This pivotal finding is widely recognized as the catalyst for the environmental justice (EJ) movement, earning Bullard the distinction of being the “father” of environmental justice scholarship.

EJ is a social movement rooted in the pursuit of equity, aiming to reform environmental policies that have historically marginalized low-income communities and communities of color. Rather than focusing solely on conservation or preservation priorities often emphasized by mainstream environmental organizations in the Global North this movement advocates for sustainable, inclusive, and community-centered development [42,57]. In the United States, the environmental justice movement distinguishes itself by addressing systemic inequalities in environmental governance. Its emphasis on justice over traditional conservation has led some scholars to question whether the goals of social equity and environmental sustainability are always aligned [52,53].

People in low-income BIPOC communities began the environmental justice movement. The movement dates back to the late 1970s when protests took place against the government in Warren County, North Carolina. The protest was against the disposal of soil containing some polychlorinated biphenyls (PCB) in a region that was mostly inhabited by Black communities PCB is a carcinogenic chemical, meaning it increases the risk of cancer [41,45].

The EJ movement in the USA is the outcome of the ‘struggle of low-class, often black communities against the incinerators and toxic waste dumps that, by accident and frequently by design, come to be sited near them (and away from affluent neighborhoods) [61]. The movement is often seen to be in contrast to the more well-known environmentalism of the middle-class Americans who have shown less concern with the disproportionate burden of toxic wastes and risks on minority communities [45,49]. Hofrichter [62] has argued that these minority communities have been unfairly at the receiving end of ‘unregulated, often racist, activities of major corporations who target them for high technology industries, incinerators and waste. Although the contexts and their historical development are different, the grassroots, activist nature of this movement has much in common with many similar movements for social justice in the developing world, whether prefixed by sustainable development or not.

The EJ movement has largely emerged as a U.S. based phenomenon. Terms such as “environmental racism” and “*environmental equity*” are often used interchangeably with EJ, though each carries distinct connotations. In the U.S. context, scholars have argued that race is a more reliable predictor than income when assessing the likelihood of a community being exposed to environmental hazards [57]. The term “*environmental racism*” was coined by Benjamin Chavis, then head of the United Church of Christ’s Commission on Racial Justice [51]. The Commission’s landmark study, Toxic Wastes and Race in the United States, concluded that race was consistently the most significant factor in the siting of commercial hazardous waste facilities. In this context, environmental racism is understood as an extension of systemic racism manifesting in housing, land use, employment, and education policies and forming part of a broader structure of institutionalized inequality [33].

### 4.2. Principles of Environmental Justice

Among the many notable achievements of the environmental justice movement, the 1991 First National People of Color Environmental Leadership Summit in Washington, D.C., stands out for establishing the 17 principles of environmental justice [63] (see Table 1). The 17 principles of environmental justice emphasize the need for equitable treatment and protection of all communities, particularly marginalized groups, in environmental decision-making and policy. These principles serve as a framework for addressing environmental injustices and ensuring that all communities have a voice in decisions that affect their environment and health.

### 4.3. The Concept of Disproportionality

Communities of color and economically disadvantaged neighborhoods are disproportionately burdened by environmental pollution and associated health risks, a pattern widely recognized as environmental injustice. Adopting a disproportional perspective challenges foundational assumptions about how environmental harm is produced and interpreted, while also questioning dominant theoretical frameworks that define human-environment interactions in a postindustrial context [13,14,18,32,37]. For instance, many social science disciplines operate under the assumption that environmental harm scales proportionally with economic development. This assumption is often encapsulated in the *IPAT* equation, where *environmental impact (I) is a function of population (P), affluence (A), and technology (T)*. Originating from the Commoner [64] debate, IPAT was developed to conceptualize the anthropogenic drivers of environmental degradation.

An analysis by the U.S. Centers for Disease Control and Prevention [65] confirmed significant disparities in pesticide exposure across racial and ethnic groups. The study examined three demographic groups Mexican Americans, non-Hispanic Whites, and non-Hispanic Blacks and found that Mexican Americans and non-Hispanic Blacks had higher average blood and urine levels for 12 out of the 14 pesticides analyzed. In other words, both the average and extreme exposure levels were consistently higher in these groups compared to non-Hispanic Whites [66]. This is particularly concerning because the severity of health outcomes often correlates with the level of exposure, meaning those with the highest exposures are at the greatest risk of harm. These findings raise a critical question: why are certain racial and ethnic groups more likely to be exposed to harmful pesticides?

Traditionally, social scientists have evaluated the effectiveness of conservation programs by analyzing aggregate patterns in land user behavior. This includes examining the adoption of conservation practices, correlating environmental attitudes with demographic variables, and assessing institutional mechanisms related to land markets and resource governance [32]. However, such approaches often overlook the influence of outliers. To address this gap, we turn to the concept of disproportionality, which has been explored across three major domains of social science [14,58,67], offering a more nuanced understanding of environmental harm and responsibility.

First, research in environmental justice has long examined how adverse environmental conditions such as poor air and water quality or inequitable land use disproportionately affect marginalized communities, including racial minorities and low-income populations [63,67,68]. These studies consistently demonstrate that environmental burdens are not randomly distributed but are shaped by underlying social structures and processes. A central and ongoing debate within this field concerns the appropriate spatial scale for analyzing the relationship between environmental degradation and social organization. Scholars have questioned whether these dynamics are best understood at the neighborhood, regional, or national level, highlighting the complexity of linking environmental outcomes to patterns of social inequality [41,45,69].

Second, the concept of disproportionality is well-established in social science research, particularly in studies examining patterns of social sanctions and interactions. This body of work explores how certain minority groups are disproportionately subjected to state-imposed penalties such as police stops or searches as well as broader forms of social discrimination, including racism and sexism [56,70,71]. A subset of this research focuses on political and social actions where the consequences are not proportionate to the demographic composition of the groups involved [14,72]. One illustrative, though less commonly cited, example is Marx’s Law of Disproportionality. This principle posits economic crises and market fluctuations stem from imbalances between production and consumption. Marx argued that economic equilibrium is a myth, and that severe disproportionalities could ultimately provoke consumer unrest and rebellion.

Third, the broader field of disproportionality research in the social sciences explores how environmental impacts vary across different social groups. Ecological researchers pioneered this line of inquiry, and it is best exemplified by the concept of the “ecological footprint” a measure of the total goods and services consumed by specific populations [73]. The ecological footprint framework has been applied at various scales, including nations, industries, and households, to highlight disparities in environmental consumption and responsibility. Within this literature, disproportionality is used to examine the unequal ecological burdens borne by different groups. For example, York et al. [74] employed the ecological footprint model to evaluate competing theories such as human ecology, modernization, and political economy in explaining the relationship between industrial development and environmental degradation. More broadly, ecological footprint research underlines how certain groups disproportionately contribute to the use or misuse of environmental goods and services [75].

Recent studies such as [76,77,78,79] have identified the disproportionate impact of pesticides on marginalized communities in the United States (see Table 2). Research by Donley et al., (2022) [18] in BMC Public Health highlights the root causes of pesticide exposure, the shortcomings of current regulatory frameworks, and potential paths forward. Additional reports from Beyond Pesticides (2022) and the Center for Biological Diversity (2022) further confirm that BIPOC communities face significantly higher risks of harm from pesticide exposure compared to the general population.

### 4.4. Global Environmental and Human Health Impacts of Pesticides

#### 4.4.1. Impact of Pesticides on the Environment

Globally, the burden of pesticide exposure falls disproportionately on the poorest and most vulnerable populations, and the United States is no exception. While pesticides play a crucial role in protecting crops, they also pose serious risks to the environment, public health, and safety. Their impact extends across terrestrial ecosystems, affecting a wide range of organisms including humans, animals, and plants. Once introduced into the environment, pesticides can harm both target and non-target species [8,9,12,13,16]. Dispersion of pesticide residues in the environment and mass killings of nonhuman biota, such as bees, birds, amphibians, fish, and small mammals, were also reported. Many pesticides exhibit broad-spectrum biological activity that goes beyond their intended use, often due to the unintended effects of certain chemical compounds. These effects can alter pathogen behavior and, in some cases, intensify disease severity. Because of their toxic nature, several pesticides have been banned in many countries [5,6,81]. For example, DDT, an organochlorine, and more recently glyphosate, an organophosphorus compound, have faced widespread restrictions. However, exceptions remain: DDT is still used in parts of Africa for malaria vector control, while glyphosate continues to be applied in agriculture across Latin America and the United States. The overuse of such agrochemicals has led to the contamination of air, soil, and water systems, raising significant environmental concerns [1,2,13,15,82,83].

The widespread use and improper disposal of pesticides by farmers, institutions, and the general public have created numerous pathways for environmental contamination [1,13,15]. Pesticides can spread through various environmental media including water, air, and soil (see Table 3) making it extremely difficult to fully control their dispersion and the resulting exposure to these hazardous substances. Many pesticides, particularly those with long half-lives, can persist in the soil for extended periods. Continuous and excessive application leads to the accumulation of toxic residues, which can harm beneficial soil organisms such as earthworms and microbes. This, in turn, disrupts soil fertility and undermines the overall health of the soil ecosystem [1,2,4]. When sprayed, pesticides can also become airborne and drift into surrounding environments, contaminating nearby water bodies and soils. Their environmental effects range from subtle ecological imbalances to the outright destruction of species [8,18].

According to (WHO-FAO) [20] report, countries were asked specifically about the environmental impacts of pesticides on ecosystems and non-target organisms. Among the respondents, 30% reported collecting data on aquatic ecosystems, 23% on terrestrial ecosystems, 16% on endangered species, 14% on wildlife, and 25% on specific environmental incidents such as fish poisonings. Data collection is generally weakest in the African and Eastern Mediterranean regions. These findings reveal substantial data gaps across all regions concerning the environmental effects of pesticide use.

#### 4.4.2. Impact of Pesticides on Human Health

Worldwide, agricultural workers are prone to dire health implications due to acute and chronic exposure to pesticides [50,84]. Environmental racism contributes to a range of serious health outcomes including disease, disability, and even death particularly in low-income BIPOC communities across the United States [18,21,27,85]. In the United States, poor people and people of color experience higher cancer rates, asthma rates, mortality rates, and overall poorer health than their affluent and white counterparts. This disproportionate exposure to environmental harms in low-income, minority communities is known as “environmental injustice’’. Since the EJM’s inception in the 1960s, empirical evidence of environmental injustice along racial and socioeconomic lines has been produced time and again [25,43]. One major contributor is pesticide exposure, which can occur both directly and indirectly. Direct exposure often happens through the application of pesticides on crops, affecting the skin, eyes, mouth, and respiratory system. This can lead to acute symptoms such as headaches, irritation, vomiting, sneezing, and skin rashes. The severity of these effects depends largely on the concentration of the pesticide and the duration of exposure. Typically, the human body eliminates pesticides through excretion via urine, bile, and glandular secretions [8,18,19]. As a result, marginalized communities face a disproportionately higher risk of developing chronic health conditions. Numerous studies have examined the health impacts of pesticide exposure, particularly among workers involved in pesticide manufacturing, agricultural labor, pest control, and greenhouse operations. Documented health effects include cancer, diabetes, respiratory illnesses, neurological disorders, reproductive issues, and oxidative stress (see Table 4).

One of the most pressing concerns surrounding the distribution and use of pesticides is the risk of poisoning among exposed individuals. According to WHO-FAO [20], only 42% of responding countries worldwide maintain a centralized database on pesticide poisoning cases or related deaths, with significant regional disparities. Such databases are least common in the African and Eastern Mediterranean regions (Table 5). Access to these databases is crucial for regulatory authorities to make informed decisions. However, only 27% of responding countries reported that their pesticide regulatory authorities have access to this data (Table 5), with particularly low access in the African, Americas, and Eastern Mediterranean regions. Moreover, public awareness remains limited. Just 25% of countries indicated that data on pesticide poisoning cases are shared with the general public. This highlights an urgent need to improve both access to and dissemination of pesticide poisoning data across several regions.

To sum up, the global survey has provided up-to-date insights into the complex issue of pesticide management, which is an area that has often been overlooked in both agricultural and public health programs where pesticides are commonly used.

### 4.5. Pesticide Policy, Usage, Exposure, and Environmental Justice in the United States

#### 4.5.1. Regulatory Framework Governing Pesticide Use in the United States

In the United States, all pesticides must be registered with the Environmental Protection Agency (EPA) before they can be legally used. This registration process is often referred to as re-registration, evaluating the potential impacts of pesticides on both human health and the environment. It aims to identify and mitigate risks of concern by thoroughly reviewing factors such as the pesticide’s chemical composition, intended crop use, application rate and frequency, and guidelines for storage and disposal. The regulatory framework for pesticide uses in the U.S. is supported by five key legislative acts [47,48], which collectively ensure that pesticide products meet safety standards and are used responsibly. (1) *the Federal Insecticide, Fungicide, and Rodenticide Act*: requires all pesticides sold and distributed in the United States (including imported pesticides) to be registered by the EPA; (2) *the Federal Food, Drug and Cosmetic Act*: requires the EPA to set pesticide tolerances for all pesticides used in or on food or in a manner that will result in a residue in or on food or animal feed. A tolerance is the maximum permissible level for pesticide residues allowed in or on human food and animal feed; (3) *Food Quality Protection Act*: this act amended both FIFRA and FFDCA. The EPA must find that a pesticide poses a “reasonable certainty of no harm” before it can be registered for use on food or feed. Each pesticide registration must be reviewed at least once every 15 years; (4) *Pesticide Registration Improvement Act*: companies must pay service fees according to the category of their registration. Shorter decision review periods are provided for reduced-risk registration applications and (5) *the Endangered Species Act*: requires federal agencies to ensure that any action they authorize, fund, or carry out, will not likely jeopardize the continued existence of any listed species, or destroy or adversely modify any critical habitat for those species.

#### 4.5.2. Pesticide Usage in the United States

In the United States, the primary use of pesticides is in the agricultural sector. Each year, approximately 500 million kilograms of pesticides are applied nationwide, amounting to an estimated $10 billion in annual expenditures [2]. According to a report by Atwood and Paisley-Jones [26], the U.S. accounts for roughly 16–18% of global pesticide spending. Due to high labor costs, herbicides are widely used as a cost-effective method for weed control. Similarly, insecticides are often the only viable option for managing vectors of diseases such as malaria [3]. Within the agricultural sector, herbicides represent the largest share of pesticide expenditures (59%), followed by insecticides (14%) and fungicides (10%). The extensive use of herbicides is not limited to croplands but extends to wildlands as well. Among all active ingredients, glyphosate has been the most widely used pesticide since 2001, followed by atrazine and metolachlor-S [1,11]. Research by Wagner et al. [94] documented a significant increase in herbicide use across U.S. croplands and confirmed that glyphosate, while effective, poses potential threats not only to weeds and grasses but also to native vegetation [1,2].

#### 4.5.3. Extent and Magnitude of Disproportionate Pesticide Exposure and Impacts

Several studies such as those by Sharma et al. [1], Anjaria and Vaghela [80], and Devi et al. [10] have documented the adverse environmental and health impacts of agrochemical use (e.g., pesticides and fertilizers) across North America, with particular focus on Mexico, Canada, and the southern and western regions of the United States. These impacts disproportionately affect communities of color and those with low socioeconomic status [18,65,85]. A recent study by Donley et al. [18] found that Black and Mexican individuals in the U.S. had biomarkers for 12 harmful pesticides in their blood and urine at levels up to five times higher than those found in white residents. It also revealed that in states like California, Louisiana, Georgia, South Carolina, Tennessee, Arkansas, and Missouri, people of color make up approximately 38% of the population but account for 63% of those living near 31 pesticide-manufacturing facilities that violate environmental laws. One of the primary drivers of this disparity is the disproportionate exposure of farm workers to pesticides. As reported by The Guardian, while the U.S. Environmental Protection Agency (EPA) enforces pesticide regulations for consumers, it allows exceptions for agricultural workers. Notably, about 90% of pesticide use in the U.S. occurs on farms, and 83% of farmworkers identify as Hispanic. The US EPA [48] has confirmed the widespread environmental and public health consequences of pesticide use on BIPOC communities across various regions of the United States, as detailed below.

##### Pesticide Production and Location of Manufacturing Facilities in BIPOC Communities

Extensive research has established that chemical manufacturing, storage, and waste disproportionately impact Black, Indigenous, and People of Color (BIPOC) as well as low-income communities in the United States [18,48]. An analysis of nine U.S. cities and counties with high concentrations of hazardous chemical facilities found that residents living within a three-mile radius of these sites were significantly more likely to be African American or Latinx and living in poverty, compared to the broader city or county populations [18,37,95]. National-level data reflect similar patterns: African American and Latinx individuals living below the poverty line are more than twice as likely to reside within one mile of a hazardous chemical facility. These disparities are even more pronounced when focusing on facilities that emit the most dangerous pollutants. Despite the health risks they pose, these facilities often fail to provide meaningful employment opportunities to the communities they affect, thereby compounding the socioeconomic and environmental burdens faced by nearby residents [33,96].

Similar patterns of environmental injustice are evident in several hazardous sites. In Louisville, Kentucky, the former Black Leaf chemical facility, a Superfund site left widespread contamination in a neighborhood where 84% of residents identify as Black and 44% live below the poverty line. These figures starkly contrast with state averages of 8% and 16%, respectively. Another Superfund site, the former United Heckathorn pesticide plant, heavily polluted the harbor in Richmond, California, a city where 84% of residents are people of color [97]. These cases illustrate how hazardous chemical facilities are disproportionately located in and around low-income, BIPOC communities exposing residents to long-term health and environmental risks while reinforcing systemic inequities.

According to a study by Donley et al. [18], as of November 2021, there were 31 pesticide manufacturing facilities in the United States that the U.S. Environmental Protection Agency (EPA) had classified as being in “Significant Violation” of foundational environmental laws, including the Clean Air Act (CAA), Clean Water Act (CWA), and the Resource Conservation and Recovery Act (RCRA). Demographic analysis of the communities surrounding these facilities reveals stark socioeconomic disparities. On average, 44% of residents living within one mile of these facilities had incomes below twice the federal poverty level substantially higher than the national average of 28% and the relevant state average of 29% [18,98]. While racial and ethnic demographics varied across sites, the average percentage of BIPOC residents within one mile of these facilities was comparable to the national average. However, when compared to the relevant state averages, the disparity becomes more pronounced: 37% of residents near these facilities identified as BIPOC, compared to a 31% state average. Site-specific data revealed significant variation, with approximately half of the facilities located in areas with higher-than-average BIPOC populations and the other half with lower-than-average representation.

Regional trends were particularly notable. In states like California and across the South where the highest concentration of these facilities exists BIPOC communities are disproportionately affected. In these regions, the average BIPOC population within one mile of a pesticide manufacturing facility was 63%, compared to the national and state averages of 40% and 38%, respectively (see Table 6). This table presents demographic data for communities located near pesticide manufacturing facilities in the United States that have been cited for violations of major environmental laws. Columns 1 and 2 list the city and state where each facility is located. Column 3 provides the Facility ID, as recorded in the U.S. Environmental Protection Agency’s (*EPA*) Enforcement and Compliance History Online (*ECHO*) database. Column 4 identifies the specific environmental law(s) violated by each facility, including the Clean Water Act (*CWA*), Clean Air Act (*CAA*), and the Resource Conservation and Recovery Act (*RCRA*). Column 5 shows the percentage of residents living within one mile of each facility who do not identify as non-Hispanic white categorized here as Black, Indigenous, and People of Color (*BIPOC*). Column 6 indicates the percentage of residents within the same radius whose incomes fall below 200% of the federal poverty level. Columns 7 and 8 provide the national averages for BIPOC and low-income populations, respectively. Columns 9 and 10 present the corresponding state-level averages for each facility. The final two rows summarize the average values across all 31 facilities and across facilities located specifically in Arkansas, California, Georgia, Louisiana, Missouri, South Carolina, and Tennessee.

Among the 31 pesticide manufacturing facilities identified as being in significant violation of environmental laws, three are located in St. Joseph, Missouri. In 2021, a federal judge ordered these facilities to be transferred to third-party oversight after thousands of containers of hazardous waste stored in rusted or leaking vessels were discovered in deteriorating buildings at risk of collapse [47,48]. Federal and state lawsuits allege that rainwater had mixed with pesticide waste inside the containers, resulting in contamination of the local sewer system and the nearby Missouri River. Demographic data further highlights the implications of environmental justice in this case. Within a one-mile radius of these three facilities, the average population is 31% BIPOC and 55% low-income compared to Missouri’s state averages of 21% and 28%, respectively [98,99]. These findings reinforce a broader pattern: pesticide manufacturing facilities in significant violation of foundational environmental laws are disproportionately located in low-income communities. While the racial and ethnic composition of affected areas varies by region, the concentration of such facilities in predominantly BIPOC neighborhoods is especially pronounced in California and several Southern states.

##### Pesticide Use and Exposure in BIPOC Communities

This section highlights national trends in pesticide exposure across different demographic groups and examines how certain populations—particularly low-income communities and communities of color—bear a disproportionate share of the health and environmental risks associated with pesticide use. Research shows a strong correlation between pesticide exposure and factors such as race, ethnicity, and socioeconomic status [18,100,101].

Racial and Economic Disparities in Pesticide Use: Research by the California Environmental Protection Agency found that pesticide use represents the most significant pollution burden in terms of racial, ethnic, and income disparities exceeding the inequities associated with multiple air pollutants and other toxic releases [102]. The study revealed that nearly all pesticide applications in California occur within 60% of ZIP codes with the highest percentages of people of color. Additional research highlights similar patterns. For example, over half of all glyphosates used in California were applied in the state’s eight most impoverished counties, where 53% of residents identify as Hispanic or Latinx compared to the statewide average of 38% [18]. In 2019 alone, more than eight million pounds of pesticides linked to childhood cancers were applied in 11 California counties with a majority Latinx population (over 50%). This equated to 4.2 pounds of these pesticides used per person. In contrast, only 770,000 pounds were used in the 25 counties with the lowest Latinx populations (less than 24%), averaging just 0.35 pounds per person. Notably, both groups of counties have comparable land area and population sizes [103].

National Disparities in Pesticide Biomarker Exposure: National data show that pesticide exposure in the United States is closely linked to race, ethnicity, and socioeconomic status. A study by Nguyen et al. [104] found that African American and Mexican American individuals had significantly higher concentrations of pesticide biomarkers in their blood and urine compared to non-Hispanic white individuals who were not living in poverty. Similarly, research by Sjödin et al. [105] revealed that disparities in pesticide exposure biomarkers were greater between white women and women of color than for any of the other 16 chemical groups tested. A study conducted by the U.S. Centers for Disease Control and Prevention [65] further confirmed that metabolites of certain legacy pesticides were found at higher levels in Mexican American and African American women over the age of 40 compared to their white counterparts. In addition, Attina et al. [106] demonstrated that the health and economic burdens associated with exposure to organophosphate pesticides were disproportionately borne by non-Hispanic Black and Mexican American populations, highlighting the intersection of environmental exposure and systemic inequality. To assess pesticide exposure across a nationally representative population, we reviewed data compiled by Donley et al. [18], which includes biomonitoring results from the U.S. population between 1999 and 2016. This dataset captures concentrations of various pesticides and their metabolites in blood and urine samples collected through national health surveys. Among the 14 pesticides or metabolites detected at levels high enough to calculate a geometric mean across three demographic groups: -non-Hispanic white, non-Hispanic Black, and Mexican American only 3 (21%) were found at higher concentrations in non-Hispanic white individuals compared to the overall population average. This finding underscores the disproportionate pesticide burden borne by communities of color in the United States.

In comparison to the national average, mean urinary and serum concentrations were elevated for 8 out of 14 (57%) and 10 out of 14 (71%) pesticides/metabolites among Mexican Americans and non-Hispanic Blacks, respectively. Furthermore, 12 of the 14 pesticides/metabolites analyzed showed higher average concentrations in either non-Hispanic Blacks or Mexican Americans compared to non-Hispanic Whites. A similar pattern emerged when examining individuals with the highest exposure levels within each demographic group. Among the 35 pesticides/metabolites with reliably identified 95th percentile concentrations, the most highly exposed non-Hispanic Whites, Mexican Americans, and non-Hispanic Blacks exceeded the total population’s 95th percentile 40%, 51%, and 57% of the time, respectively. Notably, non-Hispanic Blacks or Mexican Americans had higher 95th percentile concentrations than non-Hispanic Whites for 26 of the 35 substances. These findings indicate that non-Hispanic Blacks and Mexican Americans not only tend to have higher average levels of pesticide biomarkers in urine and blood, but also that individuals with the highest exposures in these groups are more likely to experience greater pesticide burdens than their non-Hispanic White counterparts.

##### Unequal Burdens: Pesticide Risks Among Farmworkers, Children, and Low Income BIPOC Communities

Disproportionate pesticide exposure in low-income and BIPOC communities across the United States is closely linked to serious human health risks. However, the full extent of this harm often remains obscured due to systemic challenges in documenting and tracking health outcomes in underserved and overburdened populations. Research shows that many pesticides are present at concentrations associated with increased disease incidence, particularly in communities already facing structural disadvantages [83]. Emerging evidence suggests that the specific exposures disproportionately affecting BIPOC and economically marginalized groups may lead to disproportionately severe health outcomes, including acute toxicity and chronic illness.

Yet, significant barriers hinder the ability to directly connect pesticide exposure to specific health effects in these populations. For example, Poison Control Center utilization is markedly lower among BIPOC and low-income individuals, making it difficult to compare exposure-related health outcomes across racial, ethnic, and socioeconomic groups [104,107]. These challenges are even more pronounced when assessing chronic effects, which often lack the immediate, traceable symptoms seen in acute exposures. Despite these limitations, a consistent body of evidence links pesticide exposure in low-income and BIPOC communities to adverse health outcomes. This underscores a critical environmental justice issue one that demands more equitable monitoring, stronger protection, and targeted public health interventions. Table 7 below presents a geographic overview of environmental injustice, highlighting how pesticide-related health risks are disproportionately concentrated in BIPOC communities across the United States.

## 5. Structural Drivers of Pesticide Exposure in BIPOC Communities and Its Pathways

### 5.1. Structural Drivers

As explained in Section 4.1, Section 4.2 and Section 4.3, structural racism and classism are central drivers of the unequal burden of environmental pollutants. These systems rooted in historical, institutional, cultural, and behavioral practices have consistently disadvantaged BIPOC and low-income communities, resulting in significant disparities in exposure to harmful pollutants that contribute to chronic illness and premature death. Several previous studies (e.g., [18,21,22,27,46]) have identified the following driving factors as primary contributors to the observed disparities in pesticide exposure among BIPOC communities.

(i) *Legal and regulatory foundations*: Exposure disparities are deeply rooted in U.S. laws, policies, and regulatory practices that have historically failed to protect marginalized populations equitably.

Double Standards in Pesticide Safety: Regulatory frameworks often apply less stringent safety standards in contexts that disproportionately affect BIPOC communities, particularly in agricultural and industrial zones; (ii) *Inadequate worker protections*: Many BIPOC agricultural workers face insufficient safeguards against pesticide exposure, including limited access to protective equipment and health monitoring; (iii) *Neglect of executive order 12898*: Despite its mandate to prevent disproportionate environmental harm to minority and low-income populations, Executive Order 12898 has not been effectively implemented across federal agencies; (iv) *Unaddressed off-label use*: Regulatory systems often fail to account for unintended or off-label pesticide applications, which are more likely to occur in under-resourced or poorly monitored settings; (v) *Lack of training and support*: Workers and communities frequently lack adequate training on pesticide risks and safe handling practices, increasing vulnerability; (vi) *Weak post-approval oversight*: Once pesticides are approved, follow-up monitoring and enforcement are often ineffective, allowing harmful practices to persist unchecked in BIPOC communities.

### 5.2. Behavioral Patterns and Shared Responsibilities in the BIPOC Community in Addressing Pesticide Exposure in the U.S.

Behavioral patterns and culturally informed practices among BIPOC communities in the U.S. can influence how pesticide-related risks are encountered, managed, or mitigated. These behaviors often intersect with historical trauma, systemic inequities, and culturally rooted approaches to health, safety, and compliance [64,68]. Based on empirical insights from community-defined evidence practices and studies of BIPOC behavioral patterns, the following practices and influences are noteworthy (see Table 8). However, a comprehensive study published in BMC Public Health highlights that disparities in pesticide exposure among BIPOC and low-income communities are not just behavioral they are rooted in historical and systemic injustices. Regulatory gaps such as inadequate worker protections, failure to enforce environmental justice policies, and lack of post-approval monitoring perpetuate these disparities [10,11].

While the burden of pesticide exposure is rooted in systemic failures, the BIPOC community can play a vital role in community education and awareness, advocacy, safe practices, and grassroots mobilization. These efforts, combined with institutional reform, can help reduce exposure and promote environmental justice.

### 5.3. Pathways to Address Pesticide Exposure in BIPOC Communities

Several recent studies such as [9,21,22,25,124] have attempted to explain the pathways and the mechanisms to address the challenges of Pesticide Exposure in BIPOC Communities. To reduce the disproportionate burden of pesticide exposure on BIPOC communities, the following systemic actions are recommended:

Ensure that all communities regardless of race, income, or geography are protected by consistent and science-based safety standards; Implement mechanisms to accurately track and account for pesticide-related harms in environmental justice communities; Enhance regulatory safeguards for agricultural and other frontline workers, including improved enforcement of safety protocols and access to protective equipment; Address gaps in training, oversight, and enforcement that lead to misuse or unintended exposure, particularly in vulnerable communities; Prioritize protections for children and other high-risk and vulnerable groups who are more susceptible to the health impacts of pesticide exposure; Fully implement the directive to prevent disproportionate environmental harm to minority and low-income populations through all federal agency actions and conduct independent assessments of the EPA’s pesticide regulatory processes to identify and correct potential conflicts of interest or undue industry influence [49,108,125,126]. For instance the interaction between ecosystem services and environmental justice shows how both support ecological health and social equity in urban communities in St. Louis (MO, USA) [127].

Beyond legislative measures, advancing innovation and investing in the development of safer substitutes for chemical pesticides is essential for achieving sustainable agricultural practices globally by governments, research organizations, and private businesses. While only a few studies have rigorously tested these approaches using structural equation models, the existing literature offers a range of promising interventions and strategies for improved pesticide management. The adoption of modern cultivation technologies such as *integrated pest management (IPM), organic agriculture, and biotechnology* plays a critical role in reducing reliance on chemical pesticides and natural pesticides, the so-called biopesticides [12,128]. Recent studies highlight these innovations as among the most effective strategies for significantly lowering pesticide use. However, the path toward reduced pesticide dependency and enhanced ecological sustainability is complex and multifaceted. It demands coordinated efforts from scientists, policymakers, and farmers. By embracing biotechnology, precision agriculture, and regulatory reform, we can address the challenges posed by conventional pesticide practices and move toward a more sustainable agricultural future, one that protects both environmental integrity and public health.

Based on a thorough review, future research should incorporate quantitative exposure data from national databases; use GIS mapping to visualize exposure hotspots in BIPOC communities; conduct longitudinal studies to track health outcomes over time; evaluate the effectiveness of existing pesticide regulations in protecting EJ communities; Developing community-based participatory research (CBPR) models; Supporting grassroots advocacy for safer agricultural practices; and Compare international regulatory frameworks. In addition, research on the effects of interventions for structural environmental and health inequities is lacking, particularly for Black populations. Since research is conducted and funded by structures that perpetuate racism, such as academia and government, significant efforts must be made to ensure the focus of interventions and research is equitable.

## 6. Conclusions

Pesticides are widely used in modern agriculture as a cost-effective means to boost crop yield and quality, playing a vital role in ensuring global food security amid a growing population. However, pesticides, like many other environmental pollutants, pose a major environmental justice concern due to their disproportionate impact on Black, Indigenous, and People of Color (BIPOC), as well as low-income communities, exacerbating existing health and environmental inequities. Many environmental justice initiatives, studies, policies, and organizations were created with the objective of addressing and correcting the undesirable impacts of pollutants. Among others, the environmental justice movement has come a long way over the past 50 years. There is now wide recognition that all people and communities, regardless of race, color, national origin, or income, have a right to equal protection and equitable enjoyment of the benefits provided by environmental laws and regulations. Yet the increasingly well-documented reality is that people of color and low-income communities in the United States and around the world continue to shoulder the societal burden of harmful pollution such as pesticides.

The review reveals five critical insights: (i) BIPOC communities, particularly in rural and low-income areas, experience disproportionate pesticide exposure; (ii) Structural racism and classism perpetuate environmental injustice and unequal pesticide burdens; (iii) Pesticides degrade soil, reduce biodiversity, and contaminate water, amplifying health risks; (iv) Regulatory enforcement is inconsistent, leaving significant gaps; and (v) Research on cumulative and long-term effects in BIPOC communities remains limited. Overall, pesticide-related health and environmental risks are inequitably distributed, disproportionately impacting BIPOC populations due to systemic inequities and weak regulatory oversight.

Thus, this review critically examines the multifaceted impacts of chemical pesticides on the environment and human health, highlighting the urgent need for their impact on BIPOC. This review has examined whether racial and ethnic minority populations experience greater exposure to pesticides compared to majority populations and identified the specific factors that contribute to that heightened vulnerability. Our analysis reveals that these burdens are not evenly distributed; rather, they align closely with racial, ethnic, and socioeconomic lines. By focusing on one of the most widespread pollutants globally—pesticides—we explore the extent and magnitude to which disparities in exposure and health outcomes persist. This review also recognizes that structural racism and classism systems rooted in historical, institutional, cultural, and behavioral practices have systematically disadvantaged and cumulatively oppressed BIPOC communities and individuals with low income. These systemic inequities have contributed to significant disparities in exposure to environmental pollutants, many of which are linked to premature death and chronic disease. Therefore, long-term structural change is essential; there are also actionable steps that can be taken within the current regulatory system to begin addressing these injustices as well as interventions that can make existing policies more equitable and responsive to the needs of the most affected population. Addressing this issue requires coordinated efforts from all stakeholders involved in pesticide management and environmental justice including pesticide producers, scientists, policymakers, farmworkers and growers.

## Figures and Tables

**Table 1:** The seventeen principles of EJ.

1	Sacredness of Mother Earth	EJ affirms the sacredness of Mother Earth, ecological unity, and the interdependence of all species, and the right to be free from ecological destruction.
2	Mutual respect	It demands that public policy be based on mutual respect and justice for all people, free from any form of discrimination or bias.
3	Responsible use of resources	It mandates the right to ethical, balanced, and responsible uses of land and renewable resources in the interest of a sustainable planet for humans and other living things.
4	Protection from hazards	EJ calls for universal protection from nuclear testing, extraction, production, and disposal of toxic/hazardous wastes and poisons that threaten the fundamental right to clean air, land, water, and food.
5	Self-determination	It affirms the fundamental right to political, economic, cultural, and environmental self-determination of all peoples.
6	Accountability for toxins	EJ demands the cessation of the production of all toxins, hazardous waste, and radioactive materials, and that all past and current producers be held strictly accountable to the people for detoxification and containment at the point of production.
7	Participation in decision-making	It demands the right to participate as equal partners at every level of decision-making, including needs assessment, planning, implementation, enforcement, and evaluation.
8	Safe work environment	EJ affirms the right of all workers to a safe and healthy work environment without being forced to choose between an unsafe livelihood and unemployment.
9	Compensation for victims	It protects the right of victims of environmental injustice to receive full compensation and reparations for damages as well as quality health care.
10	Government accountability	EJ considers governmental acts of environmental injustice a violation of international law, the Universal Declaration on Human Rights, and the United Nations Convention on Genocide.
11	Special relationships with native peoples	It recognizes a special legal and natural relationship of Native Peoples to the U.S. government through treaties, agreements, compacts, and covenants affirming sovereignty and self-determination.
12	Historical context	The principles were drafted and adopted at the First National People of Color Environmental Leadership Summit held on 24–27 October 1991, in Washington, D.C.
13	Grassroots movement	Since their adoption, these principles have served as a defining document for the growing grassroots movement for environmental justice.
14	Cultural respect	EJ emphasizes the importance of respecting and celebrating diverse cultures, languages, and beliefs about the natural world.
15	Economic alternatives	It promotes economic alternatives that contribute to the development of environmentally safe livelihoods.
16	Political liberation	The principles aim to secure political, economic, and cultural liberation that has been denied for over 500 years of colonization and oppression.
17	Interdependence	EJ recognizes the interdependence of all communities and the need for collective action to address environmental issues.

**Table 2:** Disproportionate Exposure and Impacts of Pesticides on BIPOC Communities.

Category	Data and Insights
Occupational Exposure	“83% of U.S. farmworkers are Hispanic/Latinx. 10,000–20,000 farmworkers (mostly Latinx) fall ill annually from pesticide exposure”.
Income and Housing	“Average farmworker income is <$20,000/year; one-third lives below the poverty line. 80% of low-income housing in NY uses pesticides indoor”.
Biomarker Evidence	“12 pesticides tracked over 20 years found in Black and Mexican Americans at levels up to 5× higher than in whites”.
Environmental Proximity	“In 7 states, 38% of the population is BIPOC, but they make up 63% of residents near pesticide plants violating environmental laws”.
Children’s Exposure	“30% of pregnant Black and Dominican women in NYC had ≥8 pesticides in their bodies; 83% had at least one in umbilical cord samples”.
Regulatory Gaps	“EPA uses a cost–benefit approach for farmworkers vs. a risk-only approach for the general public”.
Export of Banned Pesticides	“U.S. exported banned pesticides to 42 countries between 2015–2019; 78% of importing countries report > 30% of workforce poisoned annually”.

Source: [14,18,27,76,77,78,79,80].

**Table 3:** Impacts of pesticides on humans, animals and ecosystems.

Impacts of Pesticides on Humans and Animals	Impacts of Pesticides on Soil Ecosystem	Impacts of Pesticides on Aquatic Ecosystem
1. Cause of chronic diseases which affect the nervous system, reproductive system, cardiovascular system, renal system and respiratory system [1,12,17] 2. Cause headaches, skin rashes, nausea, body ache, poor concentration, dizziness, cramps, panic attacks, impaired vision, birth defects, production of benign or malignant tumors, toxicity in fetus, mutations, nerve disorders, genetic changes, blood disorders, reproductive effects and endocrine disruption. In animals, pesticides cause potential carcinogens, reproductive toxins, neurotoxins and immune toxins [4,8,12].	1. Damages and reduction in soil biomass, 2. Damages in the local metabolism, 3. Contaminate the soil nutrients and cause adverse effects on humans and the environment [2,17] 4. Cause acute poisoning for microbial biomass 5. Pollute surface and water bodies 6. Decline in the soil fertility [4,17].	1. Create pollution in aquatic ecosystems and cause ecological damage, which in turn damages the natural habitat of fish in water bodies [12,17] 2. Damages to aquatic life which includes fish and plants by reducing dissolved oxygen levels, leading to changes in the physiology of aquatic life 3. Damage to aquatic plants, animals and marine populations [83].

**Table 4:** Impacts of pesticides on human health.

Health Effects	Description
Cancer	Cancer is one of the most widely prevalent diseases across the world. Direct contact with pesticides is the leading cause of cancer around the world. This is a global issue that is presently fascinating researchers from all around the world. There is rising scientific confirmation that chemical exposure, particularly pesticides, is linked with an increased occurrence of breast cancer, bladder and colon cancer, brain cancer and liver cancer [18,86,87].
Diabetes	Several studies have confirmed a link between pesticide exposure and diabetes, indicating that prolonged contact with pesticides increases diabetes risk. A significant correlation between organochlorine compounds and diabetes has been observed, as well as a similar relationship with organophosphates in type 2 diabetes risk. However, many studies are cross-sectional, which limits the reliability of the findings. Some studies suggest a notable correlation between type 2 diabetes risk and exposure to organochlorine pesticides across different populations [88,89].
Respiratory disorders:	Lung diseases such as asthma, bronchitis, organic dust lethal conditions, hypersensitivity pneumonitis, silo filler’s lung, and neuromuscular respiratory failure can result from exposure to organic dust, chemicals, and toxic gases among farm workers. Numerous studies indicate a positive correlation between asthma and pesticide exposure [90,91].
Neurological disorders:	Exposure to pesticides significantly contributes to the development of neurological syndromes. Evidence has established a correlation between pesticide exposure and the occurrence of neurological illnesses, with Parkinson’s disease (PD) and Alzheimer’s disease being the most common conditions associated with the neurotoxic effects of pesticides. Alzheimer’s disease is a type of dementia that leads to progressive memory impairment and cognitive decline due to neurodegeneration in the cerebral cortex. Parkinson’s disease is a progressive neurological disorder that primarily affects movement [40,92].
Reproductive syndromes	The link between environmental and occupational pesticide exposure has been extensively analyzed, with numerous studies confirming that exposure to pesticides can lead to fertility disorders in both females and males. Endocrine-disrupting chemicals (EDCs) can affect hormone signaling, including that of estrogens, thyroid hormones, and androgens, all of which are crucial for normal embryonic development [84,93]. Pesticides can also have adverse effects on reproductive health through various mechanisms.

**Table 5:** Data on pesticide poisoning: Presented is the % of countries answering each question positively, and the number of responding countries (or, n). Exposure is about contact, while poisoning is about harmful effects caused by that contact.

	World	African	Americas	E.Mediter’n	European	S-E.Asia	W.Pacific
Item	% n	% n	% n	% n	% n	% n	% n
Database on poisoning cases	42% 50	11% 19	56% 9	29% 7	100% 5	50% 4	83% 6
Authorities access to database	27% 49	5% 19	13% 8	14% 7	80% 5	50% 4	67% 6
Data disseminated to public	25% 51	16% 19	22% 9	14% 7	67% 6	25% 4	33% 6

Source: [20].

**Table 6:** The % BIPOC and Low-income population that reside near pesticide manufacturing facilities that have violated environmental laws compared to national and state averages.

State	City	Facility ID	Violation	1 mile from Average		National Average		State Average	
				%BIPOC	%Low Income	%BIPOC	%Low Income	%BIPOC	%Low Income
LA	Baton Rouge	110000450020	CWA	97	60	40	28	42	36
CA	Pittsburg	110000602544	RCRA	85	45	40	28	63	27
TX	Freeport	110008170237	CAA, CWA	85	75	40	28	59	31
SC	Sumter	110004940689	CWA	68	67	40	28	36	29
AR	West Helena	110000452359	CWA	87	71	40	28	28	33
TN	Memphis	110009446643	CWA	81	72	40	28	26	33
GA	Gainesville	110000527706	RCRA	74	56	40	28	48	32
LA	Saint Gabriel	110000597426	RCRA	79	57	40	28	42	36
CA	Lathrop	110000485109	CAA	67	31	40	28	63	27
GA	Ellenwood	110044280863	RCRA	93	31	40	28	48	32
TX	Alvin	110000503722	CAA	37	20	40	28	59	31
LA	Geismar	110000597364	RCRA	44	9	40	28	42	36
TX	La Porte	110000463542	RCRA	36	33	40	28	59	31
LA	Geismar	110000449765	CAA	40	9	40	28	42	36
WV	Institute	110043803676	CWA	38	56	40	28	8	33
WV	Belle	110000344182	RCRA	7	40	40	28	8	33
IA	Williamsburg	110013104684	CWA	2	26	40	28	15	25
MO	St. Joseph	110063187411	RCRA	33	56	40	28	21	28
MO	St. Joseph	110017770624	RCRA	33	58	40	28	21	28
TX	Orange	110022523982	CAA	29	36	40	28	59	31
ID	Soda Springs	110000468351	RCRA	9	27	40	28	18	27
WA	Washougal	110000491076	CWA	13	28	40	28	32	20
WV	Nitro	110000868035	CWA	4	32	40	28	8	33
MI	Midland	110027360629	RCRA	12	47	40	28	25	25
KY	Carrollton	110000379563	CWA	16	41	40	28	16	35
TN	Newport	110035828129	RCRA	18	63	40	28	16	33
MI	Midland	110043787408	CAA	12	52	40	28	25	25
NE	Mc Cook	110045745431	RCRA	7	42	40	28	22	27
MO	St. Joseph	110000443618	RCRA	28	52	40	28	21	28
WV	Kenova	110000585974	CWA	8	39	40	28	8	33
SC	Elgin	110000351930	CAA	28	30	40	28	36	29
Average for all 31 pesticide facilities				37	44	40	28	31	29
Average for facilities in AR, CA, LA, GA, MO, SC, TN				63	51	40	28	38	31

Source: Adapted from [18]. Red: % of BIPOC or people with low income is more than 5 point higher near the facility than the state or national avg; Green: % BIPOC or people with low income is more than 5 point lower near the facility than the state or national avg; Yellow: % of BIPOC or people with low income near the facility is similar to the state or national avg (≤points); Gray: Facilities located in facilities in AR, CA, LA, GA, MO, SC, TN; Violation: Clean Water Act (CWA), Clean Air Act (CAA), and the Resource Conservation and Recovery Act (RCRA).

**Table 7:** Disproportionate pesticide risks across U.S. BIPOC communities.

State/City	Negative Impacts of Pesticides
Massachusetts	In Boston, Massachusetts, a study of public housing units where 98% of residents identified as Hispanic or Black found at least two pesticides in every one of the 42 units tested, and six or more in the majority [18].
California	By extrapolating from hospital visits in California, the US EPA estimated that 10,000–20,000 agricultural workers (predominately Latinx) experience physician-diagnosed, acute illness each year in the USA due to pesticide exposure, and that number could be as high as 300,000 acute illnesses per year when accounting for workers who do not seek care from a medical facility [108]. Occupational exposure to some agricultural pesticides is associated with an increased risk of breast cancer in California Latinx women [109]. Studies on Mexican American children in a farmworker community in California found that exposure to certain pesticides in utero or after birth was associated with negative effects on attention and neurological impacts that can affect cognitive and behavioral function [110]. Nearly three out of every four children with the highest potential for pesticide exposure at school were non-Anglo, according to a study by Gray et al. [111]. A subsequent analysis of 15 agricultural counties revealed that Hispanic children were 46% more likely than white children to attend schools located within a quarter mile of pesticide applications involving chemicals of human health concern. Even more striking, Hispanic children were 91% more likely to attend schools near the highest levels of such pesticide use [112]. In Monterey County, California, farm workers had median urinary pesticide metabolite concentrations up to 395 times higher than national averages [113]. Moreover, pesticide residues were found in 85% of dust samples collected from Washington farmworker homes, and 88% of young children living in those homes had detectable pesticide metabolites in their urine highlighting the transfer of occupational exposure into the home environment [114].
Florida	In Florida, Hispanic and Haitian female farmworkers exhibited significantly higher urinary pesticide metabolite levels than those reported in nationally representative surveys [115].
Michigan	Surveillance of occupational injuries in the state of Michigan found that people who identify as Hispanic are more likely to become ill due to pesticide exposure on the job than non-Hispanics. Between 2007–2011, the rate of acute occupational pesticide-related illness and injury was 37 times higher for agricultural workers than for non-agricultural workers [78].
New York	A study on pregnant African American and Dominican women in New York City found that pesticide levels in cord plasma were negatively associated with fetal growth [114]. A study on mothers and newborns from Cincinnati found that urinary maternal levels of organophosphate metabolites were more strongly associated with decreased birth weight among Black newborns than white newborns [116,117]. A study found that pesticide use increased with housing density, with 80% of low-income public housing facilities regularly applying pesticides inside apartments and common areas [118]. In New York City, 85% of pregnant African American and Dominican women reported using pesticides in their homes, and 83% had at least one pesticide detected in umbilical cord blood samples at birth [81]. Additionally, 30% of these mothers had eight or more pesticides detected in home air samples [119]. An analysis of breast adipose tissue from women in Long Island, New York, revealed that Black women had average total pesticide concentrations approximately 10% higher than those of white women [120].
North Carolina	In North Carolina Eight-year-old Latinx children from low-income households were exposed to an average of 5.7 different pesticides over a three-month period, with exposure profiles varying between rural and urban settings. Children are especially vulnerable to environmental toxins like pesticides due to their ongoing physical and neurological development [121]. A urinary biomonitoring study of nearly 200 farmworkers in North Carolina revealed not only widespread exposure to multiple pesticide compounds, but also continuous re-exposure throughout the year [122]. Similar findings in Idaho showed that Latinx farmworkers had detectable levels of insecticide and herbicide metabolites in every urine sample tested even after the pesticide application season had ended [114].
Washington	In Washington state, over half of the students attending schools in the most agriculturally intensive counties identified as non-white compared to a statewide average of 31% [123].
Across the United States	The study by Donley et al. [18] Shows People of Color in U.S. are more likely to be harmed by pesticides due to weak regulations and lax enforcement. U.S. pesticide exposure up to 5 times greater for people of color. Key findings from the study include *Unequal exposure, Inadequate protections for farmworkers; Environmental injustice hotspots and Toxic housing conditions*.

**Table 8:** Behavioral Patterns of BIPOC and Implications for Pesticide Exposure.

Behavioral Category	BIPOC-Specific Patterns	Implications for Pesticide Exposure
Home-based practices	Multigenerational storage, kin-taught methods	Potential for improper storage increased exposure risk
Community knowledge	Reliance on informal guidance	May conflict with regulated handling, yet culturally trusted
Distrust of authority	Skepticism of regulations or inspectors	Avoidance of official compliance, underreporting exposure
Protective modifications	Natural pest control, selective chemical use	Reduces exposure but may introduce inconsistent use or mislabeling
Language and cultural barriers	Limited English Proficiency individuals, cultural interpretations of safety	Misunderstanding instructions, improper disposal

Source: Adapted from [18,28,68].

## Data Availability

No new data were created or analyzed in this study.

## References

[1] Sharma A., Kumar V., Shahzad B., Tanveer M., Sidhu G.P.S., Handa N., Kohli S.K., Yadav P., Bali A.S., Parihar R.D. (2019). Worldwide pesticide usage and its impacts on ecosystem. SN Appl. Sci..

[2] Tudi M., Ruan H.D., Wang L., Lyu J., Sadler R., Connell D., Chu C., Phung D.T. (2021). Agriculture Development, Pesticide Application and Its Impact on the Environment. Int. J. Environ. Res. Public Health.

[3] Wanner N., DeSantis G., Alcibiade A., Tubiello F.N. (2022). Pesticides Use, Pesticides Trade and Pesticides Indicators: Global, Regional and Country Trends, 1990–2020.

[4] Garud A., Pawar S., Patil M.S., Kale S.R., Patil S. (2024). A Scientific Review of Pesticides: Classification, Toxicity, Health Effects, Sustainability, and Environmental Impact. Cureus.

[5] Mengistie B.T., Mol A.P., Oosterveer P. (2016). Private Environmental Governance in the Ethiopian Pesticide Supply Chain: Importation, Distribution and Use. NJAS-Wagening. J. Life Sci..

[6] Mengistie B.T., Mol A.P.J., Oosterveer P. (2015). Pesticide use practices among smallholder vegetable farmers in Ethiopian Central Rift Valley. Environ. Dev. Sustain..

[7] Mengistie B.T. (2021). Ethiopia: The Environmental Aspects of Policy and Practice in the Ethiopian Floriculture Industry. Environ. Policy Law.

[8] Zhou W., Li M., Achal V. (2025). A comprehensive review on environmental and human health impacts of chemical pesticide usage. Emerg. Contam..

[9] Donley N. (2019). The USA lags behind other agricultural nations in banning harmful pesticides. Environ. Health.

[10] Lynch E.E., Malcoe L.H., Laurent S.E., Richardson J., Mitchell B.C., Meier H.C. (2021). The legacy of structural racism: Associations between historic redlining, current mortgage lending, and health. SSM-Popul. Health.

[11] Brookings (2024). Research: US Pesticide Regulation Is Failing the Hardest-Hit Communities. It’s Time to Fix It. https://www.brookings.edu/articles/us-pesticide-regulation-is-failing-the-hardest-hit-communities-its-time-to-fix-it/.

[12] Daraban G.M., Hlihor R.-M., Suteu D. (2023). Pesticides vs. Biopesticides: From Pest Management to Toxicity and Impacts on the Environment and Human Health. Toxics.

[13] Yuan H., Jang J.-C., Long S., Zhu Y., Wang S., Xing J., Zhao B. (2024). A Multi-Pollutant Air Quality Analysis with Environmental Justice Considerations: Case Study for Detroit. Sustainability.

[14] Cannon C.E.B. (2024). Critical Environmental Injustice: A Case Study Approach to Understanding Disproportionate Exposure to Toxic Emissions. Toxics.

[15] Olguín-Hernández L., Carrillo-Rodríguez J.C., Mayek-Pérez N., Aquino-Bolaños T., Vera-Guzmán A.M., Chávez-Servia J.L. (2024). Patterns and Relationships of Pesticide Use in Agricultural Crops of Latin America: Review and Analysis of Statistical Data. Agronomy.

[16] Ahmad M.F., Ahmad F.A., Alsayegh A.A., Zeyaullah M., Al Shahrani A.M., Muzammil K., Saati A.A., Wahab S., Elbendary E.Y., Kambal N. (2024). Pesticides impact on human health and the environment with their mechanisms of action and possible counter measures: Review article. Heliyon.

[17] Dad K., Zhao F., Hassan R., Javed K., Nawaz H., Saleem M.U., Fatima T., Nawaz M. (2022). Pesticides Uses, Impacts on Environment and their Possible Remediation Strategies—A Review. Pak. J. Agric. Res..

[18] Donley N., Bullard R.D., Economos J., Figueroa I., Lee J., Liebman A.K., Martinez D.N., Shafiei F. (2022). Pesticides and environmental injustice in the USA: Root causes, current regulatory reinforcement and a path forward. BMC Public Health.

[19] Pathak V.M., Verma V.K., Rawat B.S., Kaur B., Babu N., Sharma A., Dewali S., Yadav M., Kumari R., Singh S. (2022). Current status of pesticide effects on environment, human health and it’s eco-friendly management as bioremediation: A comprehensive review. Front. Microbiol..

[20] WHO, FAO (2019). Global Situation of Pesticide Management in Agriculture and Public Health: Report of a 2018 WHO–FAO Survey. https://iris.who.int/bitstream/handle/10665/329971/9789241516884-eng.pdf?sequence=1.

[21] Bullard R.D. (1983). Solid Waste Sites and the Black Houston Community. Sociol. Inq..

[22] Bullard R.D. (2021). Introduction: Environmental Justice: Once a footnote, Now a headline. Harv. Environ. Law Rev..

[23] Schlosberg D. (2013). Theorising environmental justice: The expanding sphere of a discourse. Environ. Politics.

[24] Iyanda A., Boakye K., Lu Y. (2021). COVID-19: Evidenced Health Disparity. Encyclopedia.

[25] Bullard R.D., Johnson G.S. (2000). Environmental justice: Grassroots activism and its impact on public policy decision making. J. Soc. Issues.

[26] Atwood D., Paisley-Jones C. (2017). Pesticides Industry Sales and Usage 2008–2012 Market Estimates.

[27] Brulle R.J., Pellow D.N. (2006). Environmental Justice: Human Health and Environmental Inequalities. Annu. Rev. Public Health.

[28] Perea J. (2011). The echoes of slavery: Recognizing the racist origins of the agricultural and domestic worker exclusion from the National Labor Relations act. Ohio State Law J..

[29] Horst M., Marion A. (2018). Racial, ethnic and gender inequities in farmland ownership and farming in the U.S. Agric. Hum. Values.

[30] Alvarez C.H. (2022). Structural Racism as an Environmental Justice Issue: A Multilevel Analysis of the State Racism Index and Environmental Health Risk from Air Toxics. J. Racial Ethn. Health Disparities.

[31] Macias-Konstantopoulos W.L., Collins K.A., Diaz R., Duber H.C., Edwards C.D., Hsu A.P., Ranney M.L., Riviello R.J., Wettstein Z.S., Sachs C.J. (2023). Race, Healthcare, and Health Disparities: A Critical Review and Recommendations for Advancing Health Equity. West J. Emerg. Med..

[32] Morello-Frosch R., Pastor J.M., Porras C., Sadd J. (2002). Environmental justice and regional inequality in southern California: Implications for future research. Env. Health Perspect..

[33] Collins M.B., Munoz I., JaJa J. (2016). Linking ‘toxic outliers’ to environmental justice communities. Environ. Res. Lett..

[34] Curl C., Meierotto L., Som Castellano R.L. (2020). Assessment of Risk Factors for Health Disparities Among Latina Farm Workers.

[35] Egede L.E., Walker R.J., Campbell J.A., Linde S., Hawks L.C., Burgess K.M. (2023). Modern Day Consequences of Historic Redlining: Finding a Path Forward. J. Gen. Intern. Med..

[36] Claudio L. (2007). Standing on Principle: The Global Push for Environmental Justice. Environ. Health Perspect..

[37] Brodin S., Dennis G. (2024). Layers of injustice: A distributional assessment of toxic chemical facilities, releases, and cleanups. J. Environ. Manag..

[38] Nielsen J.Ø., D’haen S.A.L. (2014). Asking about climate change: Reflections on methodology in qualitative climate change research published in Global Environmental Change since 2000. Glob. Environ. Change.

[39] Cain L., Danae H.D., Christopher T., Paige W. (2024). Recent Findings and Methodologies in Economics Research in Environmental Justice. Rev. Environ. Econ. Policy.

[40] Asghar A. (2001). A Conceptual Framework for Environmental Justice Based on Shared but Differentiated Responsibilities.

[41] Taylor D. (2014). Toxic Communities: Environmental Racism, Industrial Pollution, and Residential Mobility.

[42] Sze J., London J.K. (2008). Environmental justice at the crossroads. Sociol. Compass.

[43] Sicotte D.M., Brulle R.J. (2017). Social movements for environmental justice through the lens of social movement theory. The Routledge Handbook of Environmental Justice.

[44] Ali M.A., Kamraju M. (2023). Environmental Justice and Resource Distribution. Natural Resources and Society: Understanding the Complex Relationship Between Humans and the Environment.

[45] Grineski S.E., Collins T.W., Chavez-Payan P., Jimenez A.M. (2018). Unequal distribution of environmental health risks: A conceptual framework. Soc. Nat. Resour..

[46] Kaufman J.D., Hajat A. (2021). Confronting Environmental Racism. Environ. Health Perspect..

[47] EPA-US (2022). Press Release—Judge Freezes Assets and Appoints Receiver at Pesticides Distributor in St. Joseph, Missouri.

[48] EPA-US (2023). Farmworker and Pesticides Charge to the National Environmental Justice Advisory Council 30 March 2023.

[49] Pellow D.N. (2018). What is toxic inequality? Environmental sociology as a lens to understanding environmental injustice. Annu. Rev. Sociol..

[50] Isgren E., Andersson E. (2021). An Environmental Justice Perspective on Smallholder Pesticide Use in Sub-Saharan Africa. J. Environ. Dev..

[51] Cutter S.L. (1995). Race, class and environmental justice. Prog. Hum. Geogr..

[52] Mohai P., Pellow D., Roberts J.T. (2009). Environmental justice. Annu. Rev. Environ. Resour..

[53] Shriver T.E., Webb G.R. (2009). Rethinking the scope of environmental injustice: Perceptions of health hazards in a rural native American community exposed to carbon black. Rural. Sociol..

[54] Mkutu K., Marani M., Ekitela A.L. (2019). New oil developments in a remote area: Environmental justice and participation in Turkana, Kenya. J. Environ. Dev..

[55] Schroeder R., Martin K.S., Wilson B., Sen D. (2008). Third World Environmental Justice. Soc. Nat. Resour..

[56] Serwatka T.S., Deering S., Grant P. (1995). Disproportionate Representation of African Americans in Emotionally Handicapped Classes. J. Black Stud..

[57] Čapek S.M. (1993). The “environmental justice” frame: A conceptual discussion and an application. Soc. Probl..

[58] Fraser N. (2000). Rethinking recognition. New Left Rev..

[59] Young I.M. (1990). Justice and the Politics of Difference.

[60] Solatyavari L., Anna A.K., Jeremy G. (2022). Superfund cleanup time and community characteristics: A survival analysis. J. Environ. Manag..

[61] Guha R., Martinez-Alier J. (1997). Varieties of Environmentalism: Essays North and South.

[62] Hofrichter R. (1993). Toxic Struggles: The Theory and Practice of Environmental Justice Hardcover—1 January 1993.

[63] United Church of Christ (1987). Toxic Wastes and Race in the United States: A National Report on the Racial and Socioeconomic Characteristics of Communities with Hazardous Waste Sites.

[64] Commoner B. (1972). The Environmental Cost of Economic Growth. Population. Resources and the Environment.

[65] Centers for Disease Control and Prevention/CDC (2020). National Diabetes Statistics Report.

[66] (2012). CRS Report: Pesticide Law: A Summary of the Statutes Updated RL31921 · Version 19 · 14 November 2012.

[67] Chua L., Harrison M.E., Fair H., Milne S., Palmer A., Rubis J., Thung P., Wich S., Büscher B., Cheyne S.M. (2020). Conservation and the social sciences: Beyond critique and co-optation. A case study from orangutan conservation. People Nat..

[68] Mohai P., Bryant B., Bryant B., Mohai P. (1992). Environmental racism: Reviewing the evidence. Race and the Incidence of Environmental Hazards: A Time for Discourse.

[69] Taquino M., Paris D., Gill D.A. (2002). Units of Analysis and the Environmental Justice Hypothesis: The Case of Industrial Hog Farms Wiley. Soc. Sci. Q..

[70] Holden K.C., Smock P.J. (1991). The economic costs of marital dissolution: Why do women bear a disproportionate cost?. Annu. Rev. Sociol..

[71] Washington E.M. (1996). A Survey of the Literature on Theories and Prevention of Black Male Youth Involvement in Violence. J. Negro Educ..

[72] Hill K., Leighleyv J.E. (1994). Mobilizing institutions and class representation in U.S. state electorates. Polit. Res. Q..

[73] Wackernagel M., Rees W. (1996). Our Ecological Footprint: Reducing Human Impact on the Earth.

[74] York R., Rosa E., Dietz T. (2003). Footprints on the earth: The environmental consequences of modernity. Am. Sociol. Rev..

[75] Nagy M.T., Janssens I.A., Yuste J.C., Carrara A., Ceulemans R. (2006). Footprint-adjusted net ecosystem CO_2_ exchange and carbon balance components of a temperate forest. Agric. For. Meteorol..

[76] Bradman A., Dobson C., Leonard V., Messenger B. (2010). Pest Management and Pesticide Use in California Child Care Centers.

[77] Lear L. (2002). Rachel Carson and the Awakening of Environmental Consciousness: George Washington University and National Humanities Center. https://nationalhumanitiescenter.org/tserve/nattrans/ntwilderness/essays/carson.htm.

[78] Calvert G.M., Beckman J., Prado J.B., Bojes H., Schwartz A., Mulay P., Leinenkugel K., Higgins S., Lackovic M., Waltz J. (2016). Acute occupational pesticide-related illness and injury—United States, 2007–2011. MMWR Morb. Mortal. Wkly. Rep..

[79] Epstein L. (2014). Fifty Years Since *Silent Spring*. Annu. Rev. Phytopathol..

[80] Anjaria P., Vaghela S. (2024). Toxicity of agrochemicals: Impact on environment and human health. J. Toxicol. Stud..

[81] Julien R., Adamkiewicz G., I Levy J., Bennett D., Nishioka M., Spengler J.D. (2007). Pesticide loadings of select organophosphate and pyrethroid pesticides in urban public housing. J. Expo. Sci. Environ. Epidemiol..

[82] Landrigan P.J., Claudio L., Markowitz S.B., Berkowitz G.S., Brenner B.L., Romero H., Wetmur J.G., Matte T.D., Gore A.C., Godbold J.H. (1999). Pesticides and inner-city children: Exposures, risks, and prevention. Environ. Health Perspect..

[83] Larsen A.E., Gaines S.D., Deschênes O. (2017). Agricultural pesticide use and adverse birth outcomes in the San Joaquin Valley of California. Nat. Commun..

[84] Damalas C.A., Koutroubas S.D. (2016). Farmers’ Exposure to Pesticides: Toxicity Types and Ways of Prevention. Toxics.

[85] Beyond Pesticides (2022). Highlighting the Connection: Environmental Racism and the Agricultural Industry Through History. https://beyondpesticides.org/dailynewsblog/2022/06/highlighting-the-connection-environmental-racism-and-the-agricultural-industry-through-history/.

[86] de Graaf L., Boulanger M., Bureau M., Bouvier G., Meryet-Figuiere M., Tual S., Lebailly P., Baldi I. (2022). Occupational pesticide exposure, cancer and chronic neurological disorders: A systematic review of epidemiological studies in greenspace workers. Environ. Res..

[87] Cavalier H., Trasande L., Porta M. (2022). Exposures to pesticides and risk of cancer: Evaluation of recent epidemiological evidence in humans and paths forward. Int. J. Cancer.

[88] Standl E., Khunti K., Hansen T.B., Schnell O. (2019). The global epidemics of diabetes in the 21st century: Current situation and perspectives. Eur. J. Prev. Cardiol..

[89] Chen Y., Deng Y., Wu M., Ma P., Pan W., Chen W., Zhao L., Huang X. (2024). Impact of pesticides exposure and type 2 diabetes risk: A systematic review and meta-analysis. Endocrine.

[90] Mattila T., Santonen T., Andersen H.R., Katsonouri A., Szigeti T., Uhl M., Wąsowicz W., Lange R., Bocca B., Ruggieri F. (2021). Scoping Review—The Association between Asthma and Environmental Chemicals. Int. J. Environ. Res. Public Health.

[91] Islam J.Y., Hoppin J., Mora A.M., E Soto-Martinez M., Gamboa L.C., Castañeda J.E.P., Reich B., Lindh C., Joode B.v.W.d. (2022). Respiratory and allergic outcomes among 5-year-old children exposed to pesticides. Thorax.

[92] Kamel F., Hoppin J.A. (2004). Association of Pesticide Exposure with Neurologic Dysfunction and Disease. Environ. Health Perspect..

[93] Paumgartten F.J. (2020). Pesticides and public health in Brazil. Curr. Opin. Toxicol..

[94] Wagner V., Antunes P.M., Irvine M., Nelson C.R. (2016). Herbicide usage for invasive non-native plant management in wildland areas of North America. J. Appl. Ecol..

[95] White R. (2018). Life at the Fenceline: Understanding Cumulative Health Hazards in Environmental Justice Communities. Coming Clean, The Environmental Justice Health Alliance for Chemical Policy Reform and The Campaign for Healthier Solutions. https://www.ej4all.org/life-at-the-fenceline.

[96] Ash M., Boyce J.K. (2018). Racial disparities in pollution exposure and employment at US industrial facilities. Proc. Natl. Acad. Sci. USA.

[97] U.S. Census Bureau (2019). American Community Survey 5-Year Estimates. Retrieved from Census Reporter Profile Page for Census Tract 104, Kanawha, WV. https://data.census.gov.

[98] Mascarenhas M., Grattet R., Mege K. (2021). Toxic Waste and Race in Twenty-First Century America: Neighborhood Poverty and Racial Composition in the Siting of Hazardous Waste Facilities. Environ. Soc..

[99] Hoffmann M. (2022). Lawsuit: Company Continues to Leak Chemicals into Water Supply.

[100] Landrigan P.J., Fuller R., Acosta N.J.R., Adeyi O., Arnold R., Basu N., Baldé A.B., Bertollini R., Bose-O’Reilly S., Boufford J.I. (2018). The Lancet Commission on pollution and health. Lancet.

[101] United Nations Human Rights Council (2021). Report of the Special Rapporteur on the Right to Food.

[102] Cushing L., Faust J., August L.M., Cendak R., Wieland W., Alexeeff G. (2015). Racial/Ethnic Disparities in Cumulative Environmental Health Impacts in California: Evidence From a Statewide Environmental Justice Screening Tool (CalEnviroScreen 1.1). Am. J. Public Health.

[103] Weller M. (2021). There’s Something in the Air, and It Causes Childhood Cancers.

[104] Nguyen V.K., Kahana A., Heidt J., Polemi K., Kvasnicka J., Jolliet O., Colacino J.A. (2020). A comprehensive analysis of racial disparities in chemical biomarker concentrations in United States women, 1999–2014. Environ. Int..

[105] Sjödin A., Jones R.S., Caudill S.P., Wong L.-Y., Turner W.E., Calafat A.M. (2013). Polybrominated Diphenyl Ethers, Polychlorinated Biphenyls, and Persistent Pesticides in Serum from the National Health and Nutrition Examination Survey: 2003–2008. Environ. Sci. Technol..

[106] Attina T.M., Malits J., Naidu M., Trasande L. (2019). Racial/ethnic disparities in disease burden and costs related to exposure to endocrine-disrupting chemicals in the United States: An exploratory analysis. J. Clin. Epidemiology.

[107] Feldman S.R., Vallejos Q.M., Quandt S.A., Fleischer A.B., Schulz M.R., Verma A., Arcury T.A. (2009). Health care utilization among migrant Latino farmworkers: The case of skin disease. J. Rural Health.

[108] EPA-US (1992). Regulatory Impact Analysis of Worker Protection Standard for Agricultural Pesticides.

[109] Mills P.K., Dodge J.L., Bush J., Thompson Y., Shah P. (2019). Agricultural Exposures and Breast Cancer Among Latina in the San Joaquin Valley of California. J. Occup. Environ. Med..

[110] Sagiv S.K., Bruno J.L., Baker J.M., Palzes V., Kogut K., Rauch S., Gunier R., Mora A.M., Reiss A.L., Eskenazi B. (2019). Prenatal exposure to organophosphate pesticides and functional neuroimaging in adolescents living in proximity to pesticide application. Proc. Natl. Acad. Sci. USA.

[111] Gray S., Ross Z., Walker B. (2001). Every Breath You Take: Airborne Pesticides in the San Joaquin Valley Environmental Working Group. https://www.ewg.org/research/every-breath-you-take.

[112] California Environmental Health Tracking Program (2014). Agricultural Pesticide Use Near Public Schools in California.

[113] Salvatore A.L., Bradman A., Castorina R., Camacho J., López J., Barr D.B., Snyder J., Jewell N.P., Eskenazi B. (2008). Occupational behaviors and farmworkers’ pesticide exposure: Findings from a study in monterey county, California. Am. J. Ind. Med..

[114] Curl C.L., Fenske R.A., Kissel J.C., Shirai J.H., Moate T.F., Griffith W., Coronado G., Thompson B. (2002). Evaluation of take-home organophosphorus pesticide exposure among agricultural workers and their children. Environ. Health Perspect..

[115] Runkle J.D., Tovar-Aguilar J.A., Economos E., Flocks J., Williams B., Muniz J.F., Semple M., McCauley L. (2013). Pesticide risk perception and biomarkers of exposure in Florida female farmworkers. J. Occup. Environ. Med..

[116] Whyatt R.M., E Camann D., Kinney P.L., Reyes A., Ramirez J., Dietrich J., Diaz D., Holmes D., Perera F.P. (2002). Residential pesticide use during pregnancy among a cohort of urban minority women. Environ. Health Perspect..

[117] Rauch S.A., Braun J.M., Barr D.B., Calafat A.M., Khoury J., Montesano M.A., Yolton K., Lanphear B.P. (2012). Associations of Prenatal Exposure to Organophosphate Pesticide Metabolites with Gestational Age and Birth Weight. Environ. Health Perspect..

[118] Surgan M.H., Congdon T., Primi C., Lamster S., Louis-Jacques J. (2002). Pest Control in Public Housing, Schools and Parks: Urban Children at Risk.

[119] Whyatt R.M., Rauh V., Barr D.B., Camann D.E., Andrews H.F., Garfinkel R., Hoepner L.A., Diaz D., Dietrich J., Reyes A. (2004). Prenatal Insecticide Exposures and Birth Weight and Length among an Urban Minority Cohort. Environ. Health Perspect..

[120] Muscat J.E., Britton J.A., Djordjevic M.V., Citron M.L., Kemeny M., Busch-Devereaux E., Pittman B., Stellman S.D. (2003). Adipose concentrations of organochlorine compounds and breast cancer recurrence in Long Island, New York. Cancer Epidemiol Biomark Prev..

[121] Arcury T.A., Chen H., Quandt S.A., Talton J.W., Anderson K.A., Scott R.P., Jensen A., Laurienti P.J. (2021). Pesticide exposure among Latinx children: Comparison of children in rural, farmworker and urban, non-farmworker communities. Sci. Total Environ..

[122] Arcury T.A., Grzywacz J.G., Talton J.W., Chen H., Vallejos Q.M., Galván L., Barr D.B., Quandt S.A. (2010). Repeated pesticide exposure among North Carolina migrant and seasonal farmworkers. Am. J. Ind. Med..

[123] Goldman P., Brimmer J.K., Ruiz V. (2009). Pesticides in the Air—Kids at Risk: Petition to EPA to Protect Children from Pesticide Drift. Earthjustice Farmworker Justice: Written Objections to EPA’S 31 March 2014, Response to Pesticides in the air—Kids at Risk: Petition to EPA to Protect Children From Pesticide Drift. https://earthjustice.org/wp-content/uploads/2743writtenobjections5-28-14.pdf.

[124] Donley N. Lost in the Mist: How Glyphosate Use Disproportionately Threatens California’s Most Impoverished Counties. Technical Report, November 2015. https://www.biologicaldiversity.org/campaigns/pesticides_reduction/pdfs/LostInTheMist.pdf.

[125] EPA-US (1995). Environmental Justice Strategy: Executive Order 12898.

[126] (2024). Center for Biological Diversity, Lawsuit Filed over Government Failure to Protect Endangered Species from Toxic Pesticide Malathion. https://biologicaldiversity.org/w/news/press-releases/lawsuit-filed-over-government-failure-to-protect-endangered-species-from-toxic-pesticide-malathion-2024-09-09/.

[127] Stanis S.W., Piontek E., Xu S., Mallinak A., Nilon C., Hall D.M. (2024). Residents’ Perceptions of Urban Greenspace in a Shrinking City: Ecosystem Services and Environmental Justice. Land.

[128] Ray R.L., Griffin R.W., Fares A., Elhassan A., Awal R., Woldesenbet S., Risch E. (2020). Soil CO_2_ emissions from an experimental research farm: Effects of organic amendments, temperature, and rainfall. Sci. Rep..

